# Effect of physician prescribed information to the inflammatory bowel disease patients on quality of life and disease relapse: a randomised control trial

**DOI:** 10.5195/jmla.2026.2252

**Published:** 2026-07-01

**Authors:** Vahideh Zarea Gavgani, Parisa Akbari-Ekdelu, Mohammad Hossein Somi, Asghar Mohammadpoorasl, Mina Mahami-Oskouei

**Affiliations:** 1 vgavgani@gmail.com, Professor of Medical Library and Information Science, Department of Medical Library and Information Sciences, Tabriz University of Medical Sciences, Tabriz, Iran; 2 Parisa.akbari68@yahoo.com, Department of Medical Library and Information Science, School of Management and Medical Informatics, Tabriz University of Medical Sciences, Tabriz, Iran.; 3 dr.somi.m.h@gmail.com, Professor of Gastroenterology and Liver Diseases, Tabriz University of Medical Sciences, Tabriz, Iran.; 4 ampoorasl@gmail.com, Associate Professor, Department of Epidemiology and Biostatistics, Tabriz University of Medical Sciences, Tabriz, Iran; 5 mmahami24@yahoo.com, Assistant Professor, Department of Medical Library and Information Science, School of Management and Medical Informatics, Tabriz University of Medical Sciences, Tabriz, Iran.

**Keywords:** Inflammatory bowel disease, quality of life, information prescription, medical librarian, clinical trial

## Abstract

**Background:**

Inflammatory Bowel Diseases (IBD), including Ulcerative Colitis and Crohn&s Disease, are chronic and recurrent conditions that severely impact patients' quality of life and increase the risk of complications. Providing accurate and evidence-based health information is a non-pharmacological strategy that may improve disease outcomes. This study aimed to evaluate the effect of physician-prescribed information on patients' quality of life and disease Relapse.

**Methods:**

In this randomized controlled trial, 160 patients with IBD were randomly assigned to two groups. The intervention group received a structured information prescription (IP) developed by a trained medical librarian and approved by a physician, while the control group received routine oral explanations. The World Health Organization Quality of Life Questionnaire (WHOQOL) and Time to Relapse Questionnaire (TRQ) were used to assess quality of life and Relapse, respectively. Statistical analyses included t-tests, Chi-square, and Mann-Whitney tests using Stata17 software.

**Results:**

The Relapse rate in the intervention group was significantly lower than in the control group at both two months (12.5% vs. 87.5%, p=0.004) and four months (15% vs. 42.5%, P<0.001). The risk of Relapse in the control group was more than twice as high compared to the intervention group (Hazard ratio: 2.1; 95% CI: 1.6–2.8). The mean overall quality of life scores showed an improvement in the intervention group, while a decline was observed in the control group. A significant improvement was also indicated in all quality-of-life domains in the intervention group when compared to the control group (P < 0.001).

**Conclusion:**

Physician-prescribed information interventions significantly enhance quality of life and reduce disease Relapse in IBD patients, offering a promising complementary approach in clinical care.

## INTRODUCTION

Inflammatory Bowel Disease (IBD) is a group of unpredictable, chronic, and recurrent gastrointestinal disorders that present a significant health burden worldwide. The two primary types of IBD are Ulcerative Colitis (UC) and Crohn’s Disease (CD), both of which require long-term medical management and, in some cases, surgical interventions [1].

IBD imposes substantial direct and indirect costs on healthcare systems globally. In the United States, annual direct medical costs per IBD patient range from US $ 7,824 to US $41,829, with significant variation based on disease severity and treatment regimens [2]. Similarly, European studies report considerable cost heterogeneity, with estimates ranging from €2,000 to €3,500 per patient annually, driven by differences in healthcare infrastructure, disease phenotypes, and access to biologic therapies [3]. The indirect economic impact is also substantial. Studies have shown that up to 40% of patients with IBD experience work disability at some point in their lives, leading to productivity losses exceeding $4,000 per patient per year [4]. Although IBD-related mortality remains relatively low, the disease significantly contributes to disability-adjusted life years (DALYs). According to a recent Global Burden of Disease study, IBD accounted for approximately 1.3 million DALYs worldwide in 2019, highlighting its impact on quality of life [5].

This disease typically follows a relapsing course, negatively impacting the quality of life of patients. The primary factors associated with disease Relapse include low levels of knowledge, lack of disease management and follow-up, limited understanding of treatment options, and poor adherence to medications and preventive measures [6,7]. Improving patients’ knowledge about IBD may help them better understand their condition and treatment [8]. The primary goal of IBD treatment is to improve the quality of life for patients and reduce symptoms [9]. Health-related quality of life (HRQoL) is a key concern for IBD patients, and its assessment can guide improvements in treatment methods [10].

The latest consensus standards for IBD patients, published by the European Crohn's and Colitis Organization (ECCO), conclude that optimizing care quality requires providing information and education after diagnosis [11]. Therefore, information interventions, such as information prescriptions, can improve quality of life [12], accelerate recovery, reduce hospitalization durations and Relapse rates, and ultimately lower treatment costs for these patients [9]. Information prescription is the process by which a health-care provider directly “prescribes” evidence-based, tailored health information to a patient, selecting and delivering information relevant to the patient’s condition and needs, in order to support understanding, self-management, and informed decision-making [13–15].

Information interventions can inform patients about their current health status, available treatment, care and surgical options, and the associated risks and benefits, helping patients make informed and timely decisions. Such interventions can occur at any stage of the diagnostic, treatment, or care process [16].

However, research shows that many patients receive inadequate information about their condition and are often dissatisfied with the quality and quantity of information provided by their healthcare team [17]. This issue is compounded by challenges related to patient health literacy, the ability of an individual to obtain, process, and understand basic health information and services needed to make appropriate health decisions [18]. Research indicates that many patient education resources are written at a reading level that is far too advanced for the average adult, creating a significant barrier to comprehension and adherence to treatment plans [19,20] For an information intervention to be truly effective, it must be carefully tailored not only for content but also for readability, ensuring it is accessible to a wide range of patients [21].

Meanwhile, general medical information available on public resources such as websites is often too broad and does not provide tailored medical or care recommendations for patients [22, 23]. Hence, the most effective approach to delivering treatment-related information may be through an information prescription provided by a physician. However, as physicians often lack the time and expertise to search for, retrieve, and provide tailored information to patients, a collaborative approach involving the physician, clinical/medical librarian, and patient is a promising solution. This team-based strategy can ensure that the patient’s need for evidence-based, reliable, and up-to-date information is met, based on the diagnosis and prescription provided by the treating physician [24].

A review of the literature indicates that recent studies have explored the use of IP as an effective tool for improving patient engagement and outcomes in chronic diseases such as diabetes and cancer [25–28]. However, there is limited evidence on the application of such interventions in patients with IBD. This study aims to address that gap by examining the impact of evidence-based medical information prescription, directed by clinical specialists in collaboration with a medical librarian, on the quality of life of IBD patients.

## METHODS

This study was a two-arm, parallel design randomized controlled clinical trial conducted on 160 patients with IBD who were referred to the gastroenterology department of the largest educational and medical hospital in Northwest Iran (Imam Reza Hospital, Tabriz, Iran). The Gastroenterology and Hepatology Department of Imam Reza (AS) Hospital in Tabriz is an advanced medical center in Northwest Iran.

### Inclusion criteria

**Figure 1:**
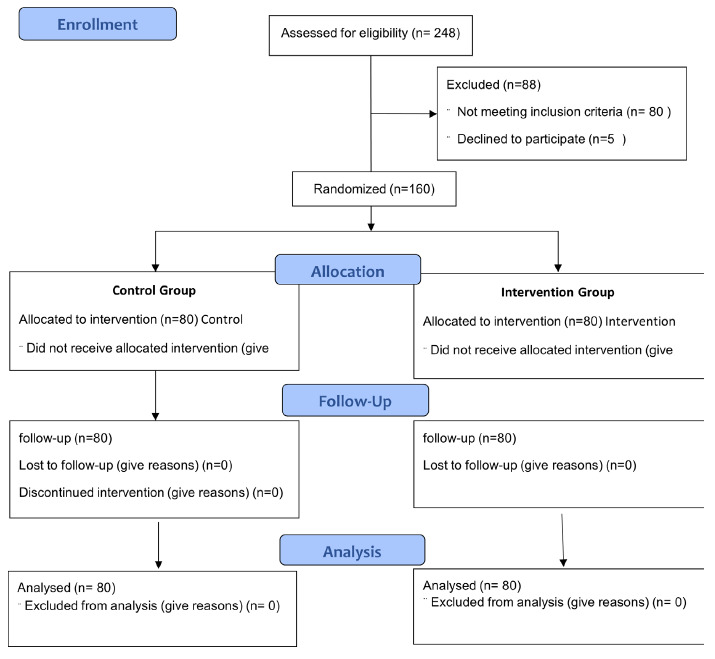
Consort Flow Diagram

The inclusion criteria were as follows: 1) a recent diagnosis of IBD (within the past 6 months), either ulcerative colitis or Crohn’s disease, 2) consent to participate in the study, 3) age between 18 and 85 years, and 4) sufficient literacy and cognitive ability to understand the explanations, instructions, and written information, either independently or with assistance from a care partner. We excluded patients who, despite having received a recent diagnosis at our clinic, had prior knowledge or experience of IBD from previous medical consultations or treatments they or their family members had received, to control for the potential confounding effect of pre-existing information exposure. Additionally, patients with other chronic conditions requiring medication were excluded to control for potential confounding effects related to comorbidities and their pharmacological treatments. Out of 248 patients referred to the gastroenterology department, 88 were excluded based on these criteria, and the study was conducted on 160 patients (Figure 1).

The study procedure was approved by the Medical Ethics Committee of Tabriz University of Medical Sciences (TBZMED.REC.1394:335). Before the commencement of the study, written informed consent was obtained from all participants or, when necessary, from their legally authorized care partner or guardian in cases where participants required assistance due to limited literacy or mild cognitive difficulties. The trial was registered in Iran's Clinical Trials Database (2N201510137612).

### Sample size

The sample size for the study was determined using the complete enumeration method, provided that the inclusion criteria were met. The patients were randomly assigned to either the intervention or control group (80 patients in each group). Randomization was performed using the “Module Ralloc” feature in Stata software. Allocation concealment was ensured using sealed, opaque, sequentially numbered envelopes containing the allocation codes. Due to the nature of the intervention, conventional blinding procedures (single or double blinding) could not be implemented for either patients or service providers. However, to minimize potential bias, a blinded assessor was used, meaning that one individual provided the intervention, while another conducted the assessments without prior knowledge of the intervention or its content. The reporting of this trial followed the CONSORT guidelines (see Appendix A file).

### Intervention

The information intervention consisted of evidence-based health and medical information prescribed by the gastroenterologists involved in the patients’ treatment. A medical librarian, trained in providing information prescriptions, was responsible for preparing the materials. The content was developed in accordance with the standards of the American Medical Association and the National Institutes of Health, ensuring a readability level appropriate for 5th and 6th-grade readers [29, 30]. The Flesch-Kincaid Grade Level formula [31] was employed to evaluate the readability of the intervention materials. Flesch-Kincaid Grade formula classifies the reading level of materials based on three levels: low (1st–5th grade), intermediate (6th–11th grade), and advanced (12th–18th grade). The core of our readability strategy, however, was the manual simplification and translation of the content by a trained medical librarian to ensure clinical accuracy and patient-friendly language. The translated information materials were carefully revised to achieve a readability level corresponding to the 5th to 6th-grade range, with the aim of enhancing comprehension and accessibility among patients with varying literacy skills. The materials were sourced from Cochrane Patient Summaries, MedlinePlus, and UpToDate databases, adhering to rigorous standards for referencing and avoiding commercial bias for example, promotional content or brand-specific drug information, with the goal of aiding neutral, evidence-based decision-making [32].

A comprehensive, multi-step information prescription process was then undertaken. The initial translation into Persian and subsequent simplification were performed manually by the medical librarian, a crucial step to ensure both linguistic accuracy and adherence to the specified readability standards. No online or machine translation tools were used during this process to maintain the integrity of the medical information and to avoid the inaccuracies and biases inherent in automated translations. The translated materials were then formatted and personalized for the patients.

The Information Prescription used in this intervention followed a standardized, four-part structure. The first section, entitled Information Prescription, included administrative and identification details: patient’s name, health condition, reason for issuance, treating physician’s name, and the medical librarian(s) responsible for content preparation. The second and main section, the Core Content, formed the educational core. It consisted of structured textual information, supplemented with simple visuals when needed (e.g., anatomical diagrams or charts), covering basic disease background and tailored, evidence-based recommendations. Typical elements included disease definition, etiology and mechanisms, treatment options, the physician’s recommended treatment with supporting evidence, and detailed self-care and follow-up instructions. The third section provided contact and follow-up information (phone number and email). Finally, the Sources and Evidence section listed all references and evidence-based sources used, ensuring scientific credibility and transparency. All these actions were performed by the trained medical librarian.

**Figure 2:**
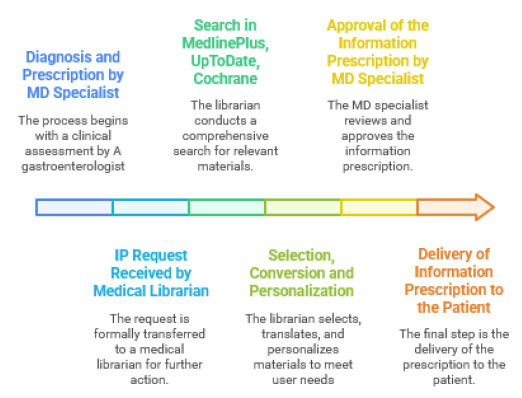
Process of Preparing and Delivering the Information Prescription for IBD Patients

The time required to prepare each Information Prescription varied based on the complexity and nature of the physician's request. For requests involving readily available background information in Farsi, the process was relatively fast, typically taking 8 to 15 minutes. In these cases, the librarian primarily focused on formatting, simplification, and adjusting readability before inserting the content into the standard template. However, preparing foreground information requiring evidence-based resources demanded a longer workflow. This process, which involved retrieving and translating evidence from reliable sources (such as Cochrane Patient Summaries or UpToDate), simplifying the content, and formatting it, usually took 30 to 45 minutes. This could extend up to two hours if complex illustrations or anatomical images required external editing and translation of labels into Farsi. In cases where a previously prepared prescription existed for a similar condition, the process was substantially accelerated, requiring only 1–2 minutes for customization and physician confirmation. The finalized version of every Information Prescription was subsequently sent to the specialist physician for final approval. The intervention group received these materials after the materials have been approved by the treating gastroenterologist, while the control group received no additional evidence-based health or medical information beyond regular verbal information from the gastroenterologist or a nurse during regular visits (Figure 2).

### Outcomes

The outcomes were assessed using the World Health Organization Quality of Life (WHOQOL) questionnaire [33], and The Time to Relapse Questionnaire (TRQ) [34]. WHOQOL assesses four domains of quality of life: physical health, psychological health, social relationships, and environment, using 24 questions (7, 6, 3, and 8 questions, respectively, for each domain). Two additional questions evaluate overall health status and quality of life but do not belong to any specific domain, bringing the total number of questions to 26. The data were coded and scored according to the standard guidelines of the WHOQOL. Scores for each domain ranged from 4 to 20, where a score of 4 indicated the poorest quality of life and a score of 20 represented the highest quality of life. Scores for each domain were calculated according to the WHOQOL standard procedure and then transformed linearly to a 0–100 scale to standardize the results across domains and simplify interpretation, with higher scores indicating better quality of life. The reliability of this questionnaire has been validated in the Iranian population [35], with Cronbach’s alpha values of 0.77 and 0.85 for different domains (α = 0.77‏و‏ α = 0.85). This questionnaire was administered twice: at the beginning of the study (in-person) and four months after the intervention (via telephone or scheduled visits).

TRQ was used, with modification, to assess the relapse, the time delay between each Relapse by asking directly from the patients and from the patients’ medical records. TRQ is a general instrument designed for chronic disease settings, but in this study, it was employed with specific adaptations to align with the clinical definition of IBD relapse and the Iranian population. The modifications were primarily focused on Cultural and Linguistic Adaptation (Localization) to enhance clarity, and Specialized Content Adaptation (Contextualization) to focus the questions on specific clinical relapse indicators relevant to the disease under study. To ensure the adequacy of the adapted instrument, both qualitative and quantitative validation procedures were executed. The content and face validity were initially established through a review by an expert panel of gastroenterological specialists. Following this, a pilot study was conducted on an independent sample (n=20) to assess the internal consistency. The resulting Cronbach's alpha for the core relapse domains was calculated to be 0.79, confirming acceptable internal reliability for its use in this population. This questionnaire was followed up twice: once at two months and again at four months after the information prescription intervention.

### Statistical analysis

Descriptive statistics include relative frequency, means, and standard deviations. The normality of the distribution of quantitative variables was examined using the Kolmogorov-Smirnov test. At the inferential level, bivariate analysis was conducted using both parametric and non-parametric tests. Disease Relapse was categorized as a binary variable (Relapse: yes/no), and the Chi-square test was applied to compare its frequency between the two groups. Mann-Whitney U tests and independent t-tests were used to compare quality of life between the groups. Independent t-tests were used when the data followed a normal distribution, while Mann-Whitney U tests were applied for non-normally distributed data. The Kaplan-Meier survival curve was utilized to compare the primary objective, namely disease Relapse, between the control and intervention groups. Hazard ratios and numbers needed to treat (NNT), along with 95% confidence intervals, were calculated. The confidence intervals were estimated using the exact method. Cox regression analysis was used to understand the potential effects of confounding variables, such as age, sex and literacy level on the disease’s Relapse. A p-value of less than 0.05 was considered statistically significant at all stages of the analysis. Data analysis was carried out using Stata software, version 17.

## RESULTS

The mean age of the participants was 37.58 ± 13.32 years, with an age range of 18 to 80 years. The mean age in the intervention group was 34.48 ± 13.52 years, while in the control group, it was 40.67 ± 12.45 years (p<0.003). Table 1 shows a comparison of the demographic characteristics of the patients in both groups. As observed in the table, there were no statistically significant differences between the two groups in any of the demographic variables, indicating that both groups were similar in terms of baseline characteristics except age of subjects.

**Table 1:** Baseline Demographic Characteristics of Patients in the intervention and control groups

Variable		Intervention (n%)	Control (n%)	Total (n%)	p-value Chi-square
Gender	Female	43 (53.75%)	34 (42.5%)	77 (48.1%)	0.206
	Male	37 (46.25%)	46 (57.5%)	83 (51.9%)	
Marital Status	Single	23 (28.75%)	16 (20%)	39 (24.4%)	0.269
	Married	57 (71.25%)	64 (80%)	121 (75.6%)	
Education Level	Less than High School education	22 (27.5%)	32 (40%)	54 (33.8%)	0.217
	High School education	19 (23.8%)	18 (22.5%)	37 (23.1%)	
	Higher Education	39 (48.8%)	30 (37.5%)	69 (43.1%)	
Disease Type	Ulcerative Colitis	78 (97.5%)	79 (98.8%)	157 (98.1%)	0.560
	Crohn's Disease	2 (2.5%)	1 (1.3%)	3 (1.9%)	

### Comparison of Disease Relapse Rate in the Intervention and Control Groups

As seen in Table 2, two months after the information prescription, the Relapse rate in the intervention group (12.5%) was significantly lower than in the control group (31.3%) (p= 0.004)

**Table 2:** Disease Relapse Rate in the Intervention and Control Groups Two Months and Four Months After the Information Prescription

Follow-up Period	Control Group Relapse Rate (n/N)	Intervention Group Relapse Rate (n/N)	P-value (Group Comparison)	Relative Risk (RR) (95% CI)	Number Needed to Treat (NNT)
Two Months Post-Intervention	31.3%	12.5%	0.004	2.5 (1.3 – 4.9)	4.5
Four Months Post-Intervention	42.5%	15.0%	<0.001	2.8 (1.6 – 5.1)	3.6

The likelihood of relapse in the first follow-up was 2.5 times higher in the control group compared to the intervention group, with a 95% confidence interval for relative risk ranging from 1.3 to 4.9. The NNT was 4.5.

Over the course of four months, 28.8% of patients (46/160) experienced a relapse of the disease. Of these, 42.5% were in the control group (34/80), and 15% were in the intervention group (12/80). This difference was statistically significant (p<.001).

In the second follow-up, the risk of relapse in the control group was 2.8 times higher than in the intervention group, with a 95% confidence interval for relative risk ranging from 1.6 to 5.1. The NNT indicated that for every 3.6 patients, one patient benefited from the intervention.

**Figure 3:**
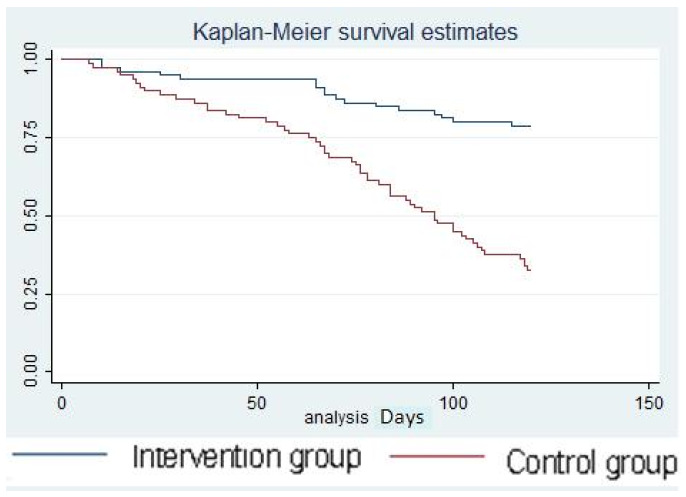
Kaplan-Meier Curve of Relapse Probability in the Two Groups

The probability of Relapse in the two groups, as shown by comparing the Kaplan-Meier survival curves in Figure 3, demonstrates that the Relapse of the disease occurs more frequently over time in the control group.

The result of a semi-parametric Cox regression analysis indicated that patients in the control group were more than twice as likely to experience a relapse compared to those in the intervention group (Hazard ratio: 2.1 (95% CI: 1.6–2.8)). The results of multivariate analysis indicated that after adjusting for potential confounding factors, the same result was observed (Hazard ratio: 1.95 (95% CI: 1.90-2.1)).

### Findings Related to the Patients' Quality of Life

Table 3 presents the comparison of the mean scores for the dimensions of the WHOQOL at the baseline and four months after the intervention in both the intervention and control groups. The mean overall quality of life scores shows an improvement in the intervention group, while the control group shows a decline. In addition, a significant improvement has been indicated in the intervention group in all quality-of-life domains when compared to the control group (See Table 3).

**Table 3:** Comparison of Quality-of-Life Dimensions Between Intervention and Control Groups (Change from Baseline to 4 Months)

Intervention				Control			
Quality of Life Dimensions	Baseline (Mean±SD)	4 Months After (Mean±SD)	Difference (Mean±SD)	Baseline (Mean±SD)	4 Months After (Mean±SD)	Difference (Mean±SD)	p-value*
Physical Health	53.71±19.73	56.91±20.94	−3.24±17.23	54.65±18.93	38.30±17.90	−16.45±16.06	P < 0.001
Psychological Health	55.14±18.43	62.16±21.18	−5.79±16.43	56.85±20.27	41.16±19.05	−15.84±16.39	P < 0.001
Social Relationships	62.13±22.33	63.75±21.38	−2.77±18.04	59.37±22.44	41.16±19.65	−17.98±18.03	P < 0.001
Environmental	59.41±16.19	64.36±20.57	−4.81±19.35	59.85±14.73	43.86±16.77	−16.29±15.38	P < 0.001
General Health	58.22±23.84	63.9±24.03	−6.17±19.18	62.18±24.26	45.78±24.51	−16.45±16.93	P < 0.001
Total Score	57.69±16.31	62.68±19.35	−5.44±14.57	57.33±16.88	39.88±16.26	−17.87±15.74	P < 0.001

* Comparison of the changes in quality-of-life score between the two groups with independent t-test and Mann-Whitney U test.

## DISCUSSION

This RCT was conducted to investigate the impact of information prescription on quality of life and disease Relapse among IBD patients. The results of this study clearly indicate that delivering an information prescription directed by treating gastroenterologist and prepared by a medical librarian from evidence-based information, can substantially enhance the quality of life in patients suffering from IBD. This positive impact is reflected in the significant reduction of Relapse rates, as well as improvements of all quality-of-life dimensions, observed in the intervention group four months after the intervention when compared to the control group.

The most striking outcome of this study is the significant difference in Relapse rates between the intervention and control groups. These findings are consistent with previous research highlighting the role of patient education and empowerment in managing chronic diseases like IBD [8]. Although a significant difference in age was observed between the groups at baseline, a multivariate analysis was performed to adjust for this and other potential confounding variables, such as disease severity. This robust analysis confirmed that the positive impact of the information intervention on Relapse rates and quality of life was independent of these factors. Most patients acknowledge the significance of clinical information and its influence on their quality of life and daily functioning. Consequently, they actively seek to acquire more knowledge about their condition. However, they often lack comprehensive education regarding their disease and advanced care plans [17,36]. Hasson’s study [37], which explored the relationship between information provision and health-related quality of life, anxiety, and depression in cancer patients, revealed that patients who received more comprehensive information had better quality of life and experienced lower levels of stress and anxiety. These results align with our findings, reinforcing the idea that improved access to relevant information can significantly enhance patients' well-being and reduce psychological distress. In this study, the provision of tailored, evidence-based information empowered patients with a better understanding of their condition, treatment options, and potential complications. This, in turn, may have reduced their anxiety and helped them make informed decisions regarding medication adherence and lifestyle modifications, contributing to the lower relapse rates. Patient education programs, therefore, play a pivotal role in enhancing patient self-awareness, contributing significantly to both pharmacological and non-pharmacological interventions, as well as fostering self-management and autonomy in their healthcare [16,17].

The intervention group in this study showed significant improvements in all four dimensions of quality of life. This is an important finding because it underscores the critical role that timely, personalized information plays in chronic disease management. The improvement in physical and psychological health is particularly notable, as these two domains are often the most affected in patients with IBD due to the unpredictable and debilitating nature of the disease. Similar results were observed in Barlow et al.’s study [38], where educational self-management interventions aimed at patients with IBD led to improvements in physical health, emotional well-being, and social functioning.

The results of the current study indicate that the educational level of participants did not exert a statistically significant influence on their quality-of-life scores following the information intervention. This outcome is consistent with the findings of Angharad Vernon et al. [39], which also found no statistically significant difference in outcomes based on the parents' educational attainment among those who participated in their educational intervention sessions. It is essential to consider that this discrepancy may be attributed to variations in health literacy rather than formal educational achievement.

The results of this study are consistent with prior research, while introducing an innovative aspect by incorporating a medical librarian in the development of the information material. This collaboration ensures that the content is not only grounded in evidence-based practice but also designed to be comprehensible and patient-friendly, addressing a critical gap in healthcare where patients often receive either insufficient information or are overwhelmed by complex and inaccessible material [40]. This gap, specifically the lack of simplified, reliable, and tailored information, was directly resolved in our study by the creation of the personalized Information Prescriptions. The involvement of a medical librarian in this study was pivotal in ensuring that the information prescriptions were both evidence-based and tailored to the needs of IBD patients. Dadashi et al. [41] have shown that medical librarians, by employing advanced search and information retrieval techniques, play an essential role in accessing such evidence. In the present study, medical librarians collaborated closely with the treating gastroenterologist to curate, synthesize, and adapt high-quality, evidence-based content from the medical literature, ensuring its accuracy and relevance. The librarian’s expertise in information retrieval and knowledge translation enabled the creation of materials that were not only scientifically robust but also structured to enhance patient comprehension and engagement. This approach is consistent with Brettle et al.’s study [42], which highlights the critical role of medical librarians in bridging the gap between complex medical evidence and patient-centered communication.

A critical factor contributing to the success of the information prescriptions in this study was the emphasis on the readability of the educational materials. To ensure accessibility, the medical librarian employed established readability metrics, such as the Flesch-Kincaid Grade Level, to tailor the content to a reading level suitable for a diverse patient population. By prioritizing readability, the intervention likely enhanced patients’ ability to comprehend and apply the information, as reflected in the observed improvements in quality-of-life scores and lower relapse rates. This approach parallels the study of Song et al. [43], who demonstrated that tailored educational interventions significantly enhanced medication adherence and quality of life in patients with chronic diseases.

The methodology employed in this study, which includes the use of a blinded assessor, serves as a key strength by effectively mitigating measurement bias. Furthermore, the utilization of standardized quality-of-life assessment tools ensures the comparability of our findings with results from studies across different populations.

This study has certain limitations that should be acknowledged. First, the age difference between the two groups is among the limitations of this research, which may have influenced the outcomes. Additionally, various other confounding variables, such as socioeconomic and ideological factors, were not assessed in this study and could potentially have affected the results, thus representing further limitations. Furthermore, the follow-up period could be considered a limitation, as it was relatively short (four months). While improvements in quality of life and reduced relapse rates were observed during this time, longer-term follow-ups are necessary to determine whether these effects are sustained over time. Another notable limitation is that the study was conducted at a single center, which restricts the generalizability of the findings. Future studies should include multicenter trials with more diverse populations to validate these results across different healthcare settings and cultural contexts. Lastly, although the study demonstrated positive outcomes in terms of Relapse rates and quality of life, it did not specifically investigate how patients used the information prescriptions or how frequently they referred to them. Further research should focus on understanding patient behaviors and attitudes toward these interventions to refine and improve intervention effectiveness. Finally, the success of our team-based approach could be a model for similar interventions in other chronic diseases, paving the way for further research into the role of medical librarians in patient education and empowerment.

## CONCLUSION

This study provides strong evidence that IPs, directed by a medical specialist and curated by a health information professional, can significantly reduce relapse rates and enhance quality of life in patients with IBD. Considering the global rise in IBD prevalence, integrating such interventions into routine care could improve patient outcomes and alleviate the economic burden associated with frequent hospitalizations and relapses. Furthermore, the findings emphasize the critical role of evidence-based health information services in hospital libraries, clinics, and medical practices. They underscore the importance of health information specialists (medical librarians) in improving health outcomes by delivering accurate, comprehensible, and tailored information to meet patients’ needs. This study highlights the intersection of healthcare, information management, and patient education, advocating for the broader inclusion of IPs in clinical guidelines as a cornerstone of patient-centered care strategies. Therefore, future research may consider the IPs intervention in different gastrointestinal diseases and different populations to produce more evidence.

## Data Availability

The complete data set will be available upon request through the correspondent author.

## Supplemental Files

**Appendix A:** Checklist & Questionnaire
