# Supplementary material for: Effect of physician prescribed information to the inflammatory bowel disease patients on quality of life and disease relapse: a randomised control trial
Source: J Med Libr Assoc. 2026 Jul 14;114(3):278–89. doi: 10.5195/jmla.2026.2252 (PMC13367306; doi:10.5195/jmla.2026.2252)
Supplement: Supplementary file 1 — Appendix A: Checklist & Questionnaire [file jmla-114-3-278-s01.docx]

**Checklist for Identifying Patients with Disease Exacerbation**

**During the past two months, have you experienced any of the following?**
(Please tick “Yes” or “No” for each item.)

- Reduction in the severity of abdominal pain? ☐ Yes ☐ No
- More than ten episodes of bloody diarrhea per day? ☐ Yes ☐ No
- Bloody diarrhea accompanied by fever? ☐ Yes ☐ No
- Improvement in anemia? ☐ Yes ☐ No
- Rectal bleeding? ☐ Yes ☐ No
- Sudden weight loss? ☐ Yes ☐ No
- Increased loss of appetite? ☐ Yes ☐ No
- Skin complications (such as nodules or skin ulcers)? ☐ Yes ☐ No
- Joint inflammation or pain (e.g., knee pain, swelling, osteoporosis, skin rash)? ☐ Yes ☐ No
- Eye inflammation, redness, or swelling? ☐ Yes ☐ No
- Mouth ulcers? ☐ Yes ☐ No
- Liver inflammation? ☐ Yes ☐ No
- Overall improvement in symptoms? ☐ Yes ☐ No
- **Additional questions:**
- How many times did the disease flare before receiving the information sheet?
- What was the main cause of the flare-up? (e.g., delayed medication intake, poor dietary adherence)
- Have your symptoms worsened during the past two months? ☐ Yes ☐ No

**Checklist for Identifying Patients with Hypochondriasis**

**During the past two months, have you:**

- Felt that, in addition to your current condition, you may be suffering from another illness (self-diagnosis)? ☐ Yes ☐ No
- Believed you may have a serious disease that has not yet been diagnosed? ☐ Yes ☐ No
  *If yes, what disease do you believe you might have?* ______________________
- Experienced obsessive thoughts or fears about having a serious illness, leading to doubt about your body’s functions? ☐ Yes ☐ No
- Felt that the information sheet increased your stress or anxiety? ☐ Yes ☐ No
- Felt that the information sheet contributed to hypochondriacal concerns? ☐ Yes ☐ No
- Felt that the information sheet helped reduce your hypochondriacal concerns? ☐ Yes ☐ No
- Felt that the information sheet reduced your stress and anxiety? ☐ Yes ☐ No

**Checklist for Patients with Changes in Medication Regimen**

**During the past two months, have you experienced any of the following regarding your medication?**

- Change in the number of prescribed medications?
  ☐ Increased ☐ Decreased ☐ No change ☐ Improved adherence ☐ Switched to other drugs
- Change in dosage of medications?
  ☐ Increased ☐ Decreased ☐ No change ☐ Improved adherence ☐ Switched to other drugs
- Change in frequency/timing of medication intake?
  ☐ Increased ☐ Decreased ☐ No change ☐ Improved adherence ☐ Switched to other drugs

**Other questions:**

- Do you have any other chronic illnesses requiring medication?
- How many times did you read the information sheet in the past two months?
- Has the frequency of colonoscopy changed in the past two months?
  ☐ Increased ☐ Decreased ☐ No change ☐ Improved
- Did reading the information sheet help you manage and control your disease? ☐ Yes ☐ No

**Quality of Life Questionnaire**

**Patient Information**

- File Number: _______
- Full Name: _______
- Age: _______
- Marital Status: _______
- Education Level: _______
- Place of Residence: _______
- Contact Number: _______
- Diagnosis: _______
- Occupation: _______
- Consultation Cost: _______
- Medication Cost: _______
- Insurance Coverage for Consultation: _______
- Insurance Coverage for Medication: _______
- Insurance Type: _______
- Number of Hospitalization Days: _______

**Instructions**

This questionnaire is designed to assess your quality of life. Please read each statement carefully and, based on your feelings, experiences, and daily life during the **past four weeks**, mark the option that best represents your situation.

**General Health**

1. How would you rate your overall quality of life?
   ☐ Very poor ☐ Poor ☐ Neither poor nor good ☐ Good ☐ Very good
2. How satisfied are you with your health?
   ☐ Very dissatisfied ☐ Dissatisfied ☐ Neither satisfied nor dissatisfied ☐ Satisfied ☐ Very satisfied

**Physical Health (7 items)**

1. How often during the past two weeks did you experience increased bowel movements?
   ☐ None ☐ Very little ☐ A little ☐ Moderate ☐ Much ☐ Very much ☐ More than usual
2. How often during the past two weeks did your illness cause you to cancel or postpone tasks or social activities?
   ☐ Never ☐ Very rarely ☐ Rarely ☐ Sometimes ☐ Often ☐ Very often ☐ Always
3. How often during the past two weeks did you experience abdominal cramps?
   ☐ Never ☐ Very little ☐ A little ☐ Moderate ☐ Much ☐ Very much ☐ Always
4. Overall, how satisfied are you with your sleep quality?
   ☐ Very dissatisfied ☐ Dissatisfied ☐ Neither satisfied nor dissatisfied ☐ Satisfied ☐ Very satisfied
5. To what extent do bodily pain and physical problems prevent you from performing daily or favorite activities?
   ☐ Not at all ☐ A little ☐ Moderate ☐ Much ☐ Very much ☐ Extremely
6. To what extent do you need medical treatment (medications or special instructions) for daily activities?
   ☐ Not at all ☐ A little ☐ Moderate ☐ Much ☐ Very much ☐ Extremely
7. How satisfied are you with your ability to perform daily living activities?
   ☐ Very dissatisfied ☐ Dissatisfied ☐ Neither satisfied nor dissatisfied ☐ Satisfied ☐ Very satisfied

**Psychological (6 items)**

1. How much do you enjoy life?
   ☐ Not at all ☐ A little ☐ Moderate ☐ Much ☐ Very much ☐ Extremely
2. To what extent is your life meaningful and valuable?
   ☐ Not at all ☐ A little ☐ Moderate ☐ Much ☐ Very much ☐ Extremely
3. How well are you able to concentrate on your daily activities?
   ☐ Not at all ☐ A little ☐ Moderate ☐ Much ☐ Very much ☐ Extremely
4. How satisfied are you with your body image and physical appearance?
   ☐ Very dissatisfied ☐ Dissatisfied ☐ Neither satisfied nor dissatisfied ☐ Satisfied ☐ Very satisfied
5. In daily life, how much do you feel secure and at peace?
   ☐ Not at all ☐ A little ☐ Moderate ☐ Much ☐ Very much ☐ Extremely
6. How often do you experience negative emotions such as hopelessness, anxiety, or depression?
   ☐ Never ☐ Rarely ☐ Sometimes ☐ Often ☐ Very often ☐ Always

**Social Relationships (3 items)**

1. How satisfied are you with your personal relationships (e.g., family, friends)?
   ☐ Very dissatisfied ☐ Dissatisfied ☐ Neither satisfied nor dissatisfied ☐ Satisfied ☐ Very satisfied
2. How satisfied are you with your sexual life?
   ☐ Very dissatisfied ☐ Dissatisfied ☐ Neither satisfied nor dissatisfied ☐ Satisfied ☐ Very satisfied
3. How satisfied are you with the support you receive from friends and acquaintances?
   ☐ Very dissatisfied ☐ Dissatisfied ☐ Neither satisfied nor dissatisfied ☐ Satisfied ☐ Very satisfied

**Environment (8 items)**

1. How healthy and hygienic is your living environment?
   ☐ Not at all ☐ A little ☐ Moderate ☐ Much ☐ Very much ☐ Extremely
2. Do you have sufficient financial resources to meet your needs?
   ☐ Not at all ☐ A little ☐ Moderate ☐ Much ☐ Very much ☐ Completely
3. How available is the daily information and news you need?
   ☐ Not at all ☐ A little ☐ Moderate ☐ Much ☐ Very much ☐ Completely
4. How much opportunity do you have to engage in personal interests or leisure activities?
   ☐ Not at all ☐ A little ☐ Moderate ☐ Much ☐ Very much ☐ Completely
5. How satisfied are you with the conditions and facilities of your place of living?
   ☐ Very dissatisfied ☐ Dissatisfied ☐ Neither satisfied nor dissatisfied ☐ Satisfied ☐ Very satisfied
6. How satisfied are you with access to healthcare services?
   ☐ Very dissatisfied ☐ Dissatisfied ☐ Neither satisfied nor dissatisfied ☐ Satisfied ☐ Very satisfied
7. How satisfied are you with your access to transportation and commuting facilities?
   ☐ Very dissatisfied ☐ Dissatisfied ☐ Neither satisfied nor dissatisfied ☐ Satisfied ☐ Very satisfied
8. How satisfied are you with your ability to use your work capacity (e.g., shopping, walking, visiting relatives)?
   ☐ Very dissatisfied ☐ Dissatisfied ☐ Neither satisfied nor dissatisfied ☐ Satisfied ☐ Very satisfied
